# Circadian rhythm in cardiovascular diseases: a bibliometric analysis of the past, present, and future

**DOI:** 10.1186/s40001-023-01158-8

**Published:** 2023-06-24

**Authors:** Ruoning Chai, Zelin Ye, Qian Wu, Wenjing Xue, Shuqing Shi, Yihang Du, Huaqin Wu, Yi Wei, Yuanhui Hu

**Affiliations:** Guang’anmen Hospital, China Academy of Chinese Medical Sciences, Beijing, China

**Keywords:** Diurnal rhythm, Research trends, Citespace, Chronotherapy, Heart

## Abstract

**Background:**

One of the most prominent features of living organisms is their circadian rhythm, which governs a wide range of physiological processes and plays a critical role in maintaining optimal health and function in response to daily environmental changes. This work applied bibliometric analysis to explore quantitative and qualitative trends in circadian rhythm in cardiovascular diseases (CVD). It also aims to identify research hotspots and provide fresh suggestions for future research.

**Methods:**

The Web of Science Core Collection was used to search the data on circadian rhythm in CVD. HistCite, CiteSpace, and VOSviewer were used for bibliometric analysis and visualization. The analysis included the overall distribution of yearly outputs, top nations, active institutions and authors, core journals, co-cited references, and keywords. To assess the quality and efficacy of publications, the total global citation score (TGCS) and total local citation score (TLCS) were calculated.

**Results:**

There were 2102 papers found to be associated with the circadian rhythm in CVD, with the overall number of publications increasing year after year. The United States had the most research citations and was the most prolific country. Hermida RC, Young ME, and Ayala DE were the top three writers. The three most notable journals on the subject were Chronobiology International, Hypertension Research, and Hypertension. In the early years, the major emphasis of circadian rhythm in CVD was hormones. Inflammation, atherosclerosis, and myocardial infarction were the top developing research hotspots.

**Conclusion:**

Circadian rhythm in CVD has recently received a lot of interest from the medical field. These topics, namely inflammation, atherosclerosis, and myocardial infarction, are critical areas of investigation for understanding the role of circadian rhythm in CVD. Although they may not be future research priorities, they remain of significant importance. In addition, how to implement these chronotherapy theories in clinical practice will depend on additional clinical trials to get sufficient trustworthy clinical evidence.

## Introduction

Circadian rhythms are fundamental to the biology of almost all life on the planet [1]. These rhythms are driven by an internal biological clock, which is known as the circadian clock. The circadian clock is responsible for regulating a wide range of physiological processes that occur on a daily basis, including sleep–wake cycles [2], hormone secretion [3, 4], metabolism [5, 6], Immune system [7], and gene expression [8–10]. In many organisms, including humans, the circadian clock is located in the suprachiasmatic nucleus of the hypothalamus [11]. The suprachiasmatic nucleus receives input from the eyes, which allows it to synchronize the circadian clock to the external light–dark cycle. This synchronization ensures that the circadian rhythms remain aligned with the environmental cycles and are able to provide maximum survival and competitive advantage. When the body’s natural circadian rhythm is disrupted, it can lead to a range of health problems. Research has shown that disturbances to circadian rhythmicity can increase the likelihood of acute myocardial infarction [12–14], stroke [15–17], arrhythmias [18–20], and other unfavorable cardiovascular events. In addition, the circadian rhythm is recognized to affect several cardiovascular events, including endothelial function [21–23], thrombus formation [24, 25], blood pressure [26, 27], and heart rate [28]. Thus, it is not surprising that incidence of adverse cardiovascular events fluctuates depending on the time of day given the diurnal regulation. Myocardial infarctions are more likely to occur in the early morning than at night [29]. This matutinal clustering is also seen in the frequency of strokes, arrhythmias, and sudden cardiac death, as well as the rupture of abdominal aortic aneurysms [30–32].

Bibliometrics is a method for analyzing publications qualitatively and quantitatively. This method allows researchers to gain immediate insight into the thematic evolution, primary study domains, and future research paths in a certain research field [33]. Bibliometrics is now frequently employed as an auxiliary research tool in a wide range of subjects. However, there are few bibliometric studies on circadian rhythm in CVD.

In this study, we utilized bibliometric approaches to evaluate the research state, present research emphasis, and develop research trends in the field of circadian rhythm in CVD during the last two decades, highlighting potential avenues for future research.

## Materials and methods

### Data source and search strategy

**Fig. 1:**
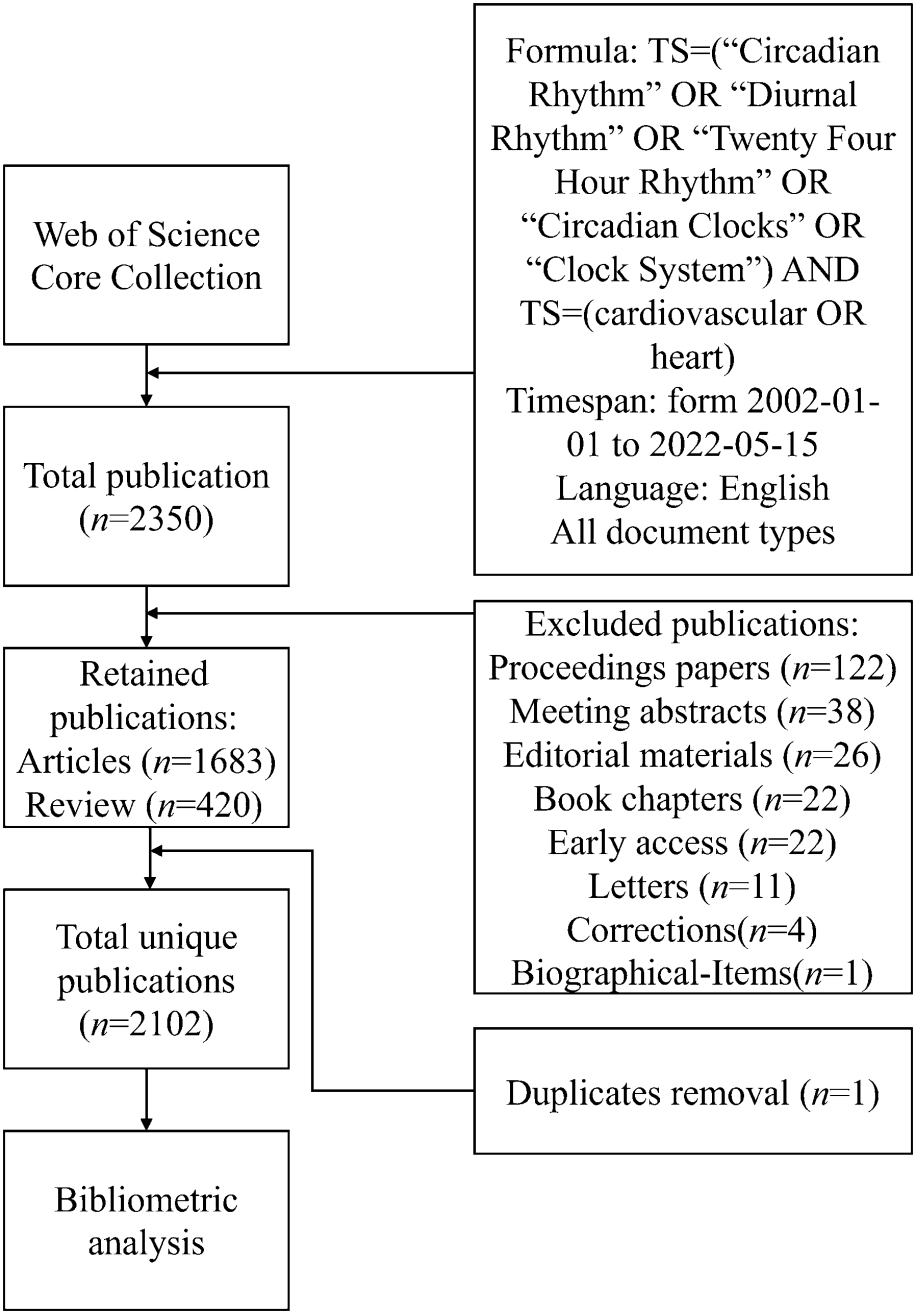
The flowchart illustrates the search formula and selection process in this study. Formula: TS = (“Circadian Rhythm” OR “Diurnal Rhythm” OR “Twenty Four Hour Rhythm” OR “Circadian Clocks” OR “Clock System”) AND TS = (cardiovascular OR heart)

All publications were from the Web of Science Core Collection (WoSCC). The data from WoSCC have the most complete data structure, including publication type (PT), author (AU), journal (SO), keyword (DE), abstract (AB), institution (CI) and reference (CR). The study used a search formula of “TS = (“Circadian Rhythm” OR “Diurnal Rhythm” OR “Twenty Four Hour Rhythm” OR “Circadian Clocks” OR “Clock System”) AND TS = (cardiovascular OR heart)”, which is described in Fig. 1 along with the inclusion criteria for the study. The following were the criteria for the publication’s inclusion: (1) research on circadian rhythm in CVD; (2) the type of publications included articles and reviews and freely available data; (3) the language of the publication was English. The following were the criteria for the publication’s exclusion: (1) the publications did not address the topic of the study; (2) the publications were news, conference abstracts, or briefs. To ensure accurate data updates, all of the aforementioned operations were completed within a 24-h period, on May 15th, 2022.

### Eligibility criteria and data collection

The document types were only articles and reviews. Duplicate studies were removed. All the information, including the number of papers and citations, publication year, titles, authors, affiliations, keywords, countries, journals, and references, was collected for bibliometric analysis.

### Statistical analysis

In this study, VOSviewer (version 1.6.18), HistCite (version 12.03.17), and CiteSpace (version 5.8.R3) were used to perform the bibliometric analysis.

VOSviewer was used to visualize complex co-citation networks [34], such as the collaboration and time trends among countries, institutions, and individuals. The size of the nodes represents the number of publications; the thickness of the line represents the strength of the link, and the colors of the nodes represent different clusters or times.

HistCite was utilized to determine the total number of publication records, total global citation score (TGCS), and total local citation score (TLCS) for each publishing year, active nations, top institutions, core journals, and authors. The TLCS refers to the number of times an article is cited in the current database, which means attracting attention from the same field. Therefore, TLCS is used to rank the authors and journals. More crucially, it was used to find the sample citation routes of relevant references [35].

CiteSpace was used to assess the visual study of the knowledge domain and developing trends [36], such as cluster analysis, dual-map citation overlay, timeline or time zone views, references, and keywords citation bursts [37]. Cluster analysis may be used to categorize references and keywords as well as highlight interesting study topics on circadian rhythmicity in CVD. In cluster analysis, the modularity *Q* and mean silhouette are two key assessment metrics. *Q* greater than 0.3 implies that the clustering structure is substantial. The presence of a mean silhouette greater than 0.5 shows that the clustering results are credible. Keywords and references bursts are frequently employed to discover new research trends in the subject [38]. It has two interpretations: (1) in keyword analysis, it represents the frequency of words or phrases used in the citing document. (2) In co-cited references analysis, it represents the frequency of citations received by the cited document.

## Results

### Overall distribution

**Fig. 2:**
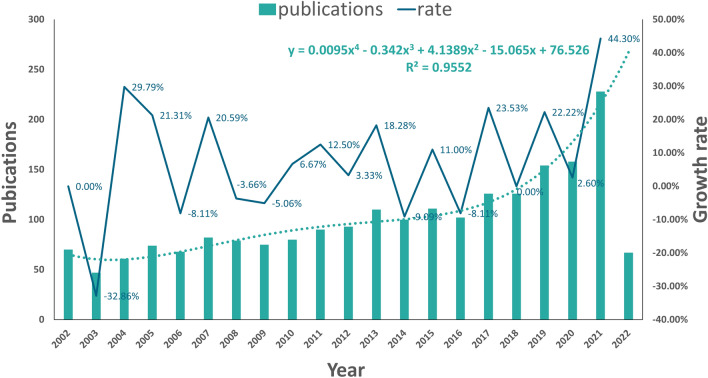
Overall distribution of annual output trends and growth rate on circadian rhythm in CVD

A total of 2102 publications related to the circadian rhythm in CVD were retrieved from WoSCC. Linear regression analysis showed that the annual number of publications on circadian rhythm in CVD has undergone an overall increasing trend since 2002 (Fig. 2). So far, the annual growth trend is in line with the fitting curve *y* = 0.0095*x*^4^ − 0.342*x*^3^ + 4.1389*x*^2^ − 15.065*x* + 76.526 (*R*^2^ = 0.9552). According to this curve, it is predicted that the number of publications in 2022 will reach about 250. These publications have been cited 62,070 times, with an average of 29.53 times per publication. The annual publication count exhibits fluctuations, however, on the whole, it is increasing and thus indicates promising prospects for research in this area.

### Countries and regions

**Fig. 3:**
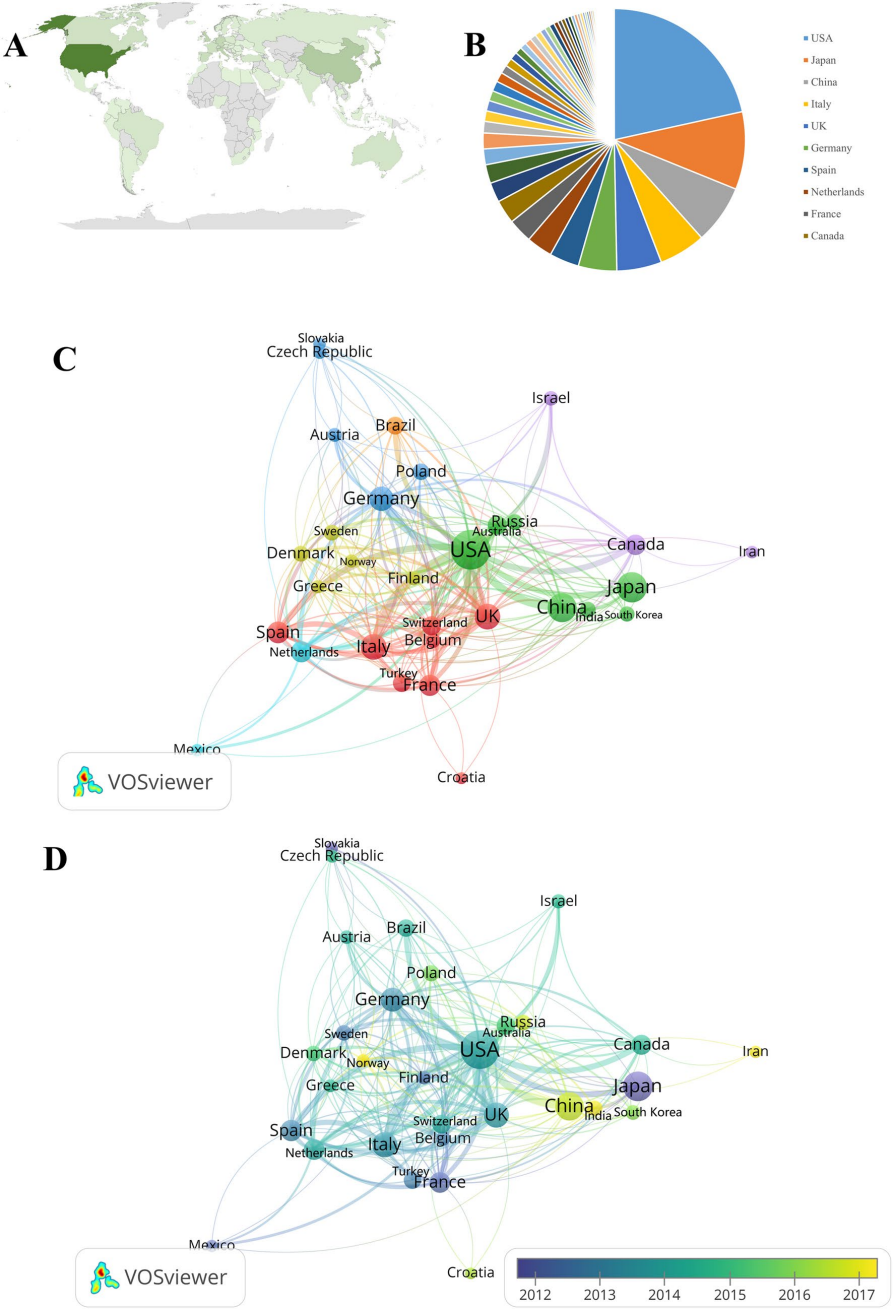
Leading countries related to circadian rhythms in CVD research. **A** Geographical distribution of global output; **B** pie chart of the countries; **C** visual cluster analysis of countries; **D** publication number by country over time

From 2002 to 2022, 80 countries and regions published research papers on circadian rhythm in CVD. The shade of green reflected the amount of countries’ publications—the darker the green, the greater the number of publication (Fig. 3A). The top 10 countries with the highest number of publications have generated about 74.59% (*n* = 1568) of the publications in the world (Fig. 3B). The United States was the most cited country for published articles (cited 28,803 times), followed by Japan (cited 7258 times), and Italy (cited 5599 times). The top three countries in centrality were the United States (centrality = 0.72), China (centrality = 0.20), and the UK (centrality = 0.19), demonstrating these countries contribution in this field (Table 1). The international collaboration network visualization showed that the collaboration between countries was relatively close. The United States has been in collaboration with almost all the other countries (Fig. 3C). From the perspective of the publication number, the USA has consistently been the top publisher (biggest node), Japan has been stable (deepest color), and China is a late starter in this field compared to other countries, but it is developing rapidly (Fig. 3D).

**Table 1:** Countries circadian rhythm in CVD-related publications

Rank	Country	Publications (*n*)	TLCS	TGCS	Average citation	Centrality
1	USA	619	1928	28,803	46.53	0.72
2	Japan	275	688	7258	26.39	0.06
3	China	209	203	2823	13.51	0.20
4	Italy	165	426	5599	33.93	0.09
5	UK	159	197	4274	26.88	0.19
6	Germany	136	460	4460	32.79	0.08
7	Spain	105	206	3268	31.12	0.01
8	Netherlands	92	103	3269	35.53	0.15
9	France	86	336	3041	35.36	0.18
10	Canada	84	90	2014	23.98	0.04

### Institutions and authors

**Fig. 4:**
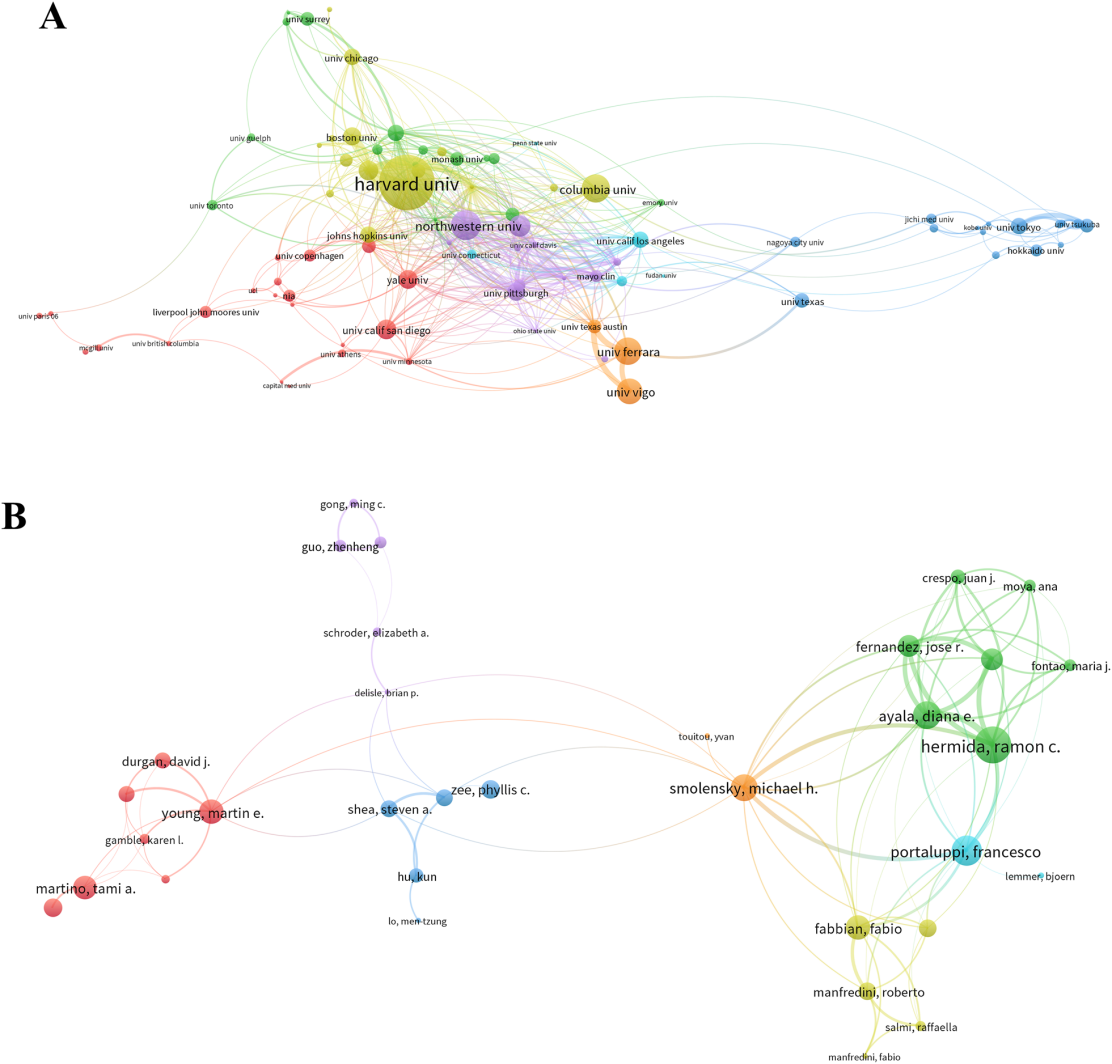
Visualization of institutes and authors collaboration analysis. (**A**–**B**, respectively) Cluster analysis of institutes and authors’ network

The top 10 institutions with the highest output on circadian rhythm in CVD research are shown in Table 2. Harvard University (*n* = 39) was the leading institution in terms of output, followed by the University of Tokyo (*n* = 35), the University of Alabama at Birmingham (*n* = 33), and the University of Ferrara (*n* = 31). Institutional collaboration was relatively high level. The Harvard University-led collaboration groups demonstrated the closest collaboration with other schools (Fig. 4A).

**Table 2:** The top 10 productive institutions related to circadian rhythm in CVD

Rank	Institution	Country	Publication counts	TGCS	TLCS	Average citation
1	Harvard Univ	USA	39	4244	307	108.82
2	Univ. Tokyo	Japan	35	929	172	26.54
3	Univ. Alabama Birmingham	USA	33	955	150	28.94
4	Univ. Ferrara	Italy	31	1766	323	56.97
5	Univ. Vigo	Spain	31	1652	361	53.29
6	Brigham & Womens Hosp.	USA	29	1209	191	41.69
7	Univ. Minnesota	USA	29	362	34	12.48
8	Harvard Med. Sch	USA	26	229	13	8.81
9	Univ. Penn	USA	26	1341	223	51.58
10	Univ. Copenhagen	Denmark	25	681	41	27.24

The top three most productive authors were Hermida RC (published 34 articles), Young ME (published 25 articles), and Ayala DE (published 24 articles). In Table 3, Hermida RC of Universidade de Vigo got the highest TLCS (score = 398), followed by Ayala DE of Universidade de Vigo (score = 287) and Portaluppi F of the University of Ferrara (score = 271).

**Table 3:** The top 10 authors with highest TLCS related to circadian rhythm in CVD

Rank	Name	Country	Affiliation	Counts	TLCS	TGCS	H-index
1	Hermida RC	Spain	Universidade de Vigo	34	398	1797	56
2	Ayala DE	Spain	Universidade de Vigo	24	287	1279	51
3	Portaluppi F	Italy	University of Ferrara	19	271	1376	58
4	Young ME	USA	University of Alabama Birmingham	25	255	1032	58
5	FitzGerald GA	USA	University of Pennsylvania	6	217	661	114
6	Mojon A	Spain	Universidade de Vigo	19	215	933	42
7	Fernandez JR	Spain	Universidade de Vigo	19	209	948	55
8	Smolensky MH	USA	University of Texas Austin	21	196	1062	47
9	Scheer FAJL	USA	Harvard Medical School	19	175	1118	58
10	Martino TA	Canada	University of Guelph	11	166	728	28

Through TLCS data, we discovered that the study of Universidade de Vigo and its researchers piqued the interest of a large number of scholars. But Fig. 4A shows that the link lines between Universidade de Vigo and other universities were insufficient and sparse, implying that Universidade de Vigo lacked collaboration with others. This result seems inharmonious, implying that there may be some concealed bias in Vigo University’s work. Their work, for example, may lack independent source data validation from non-host universities [39]. The visualization analysis of the author is shown in Fig. 4B. It is apparent that collaboration between authors from other institutions is lacking, and they prefer to collaborate inside their affiliations.

### Journals

**Fig. 5:**
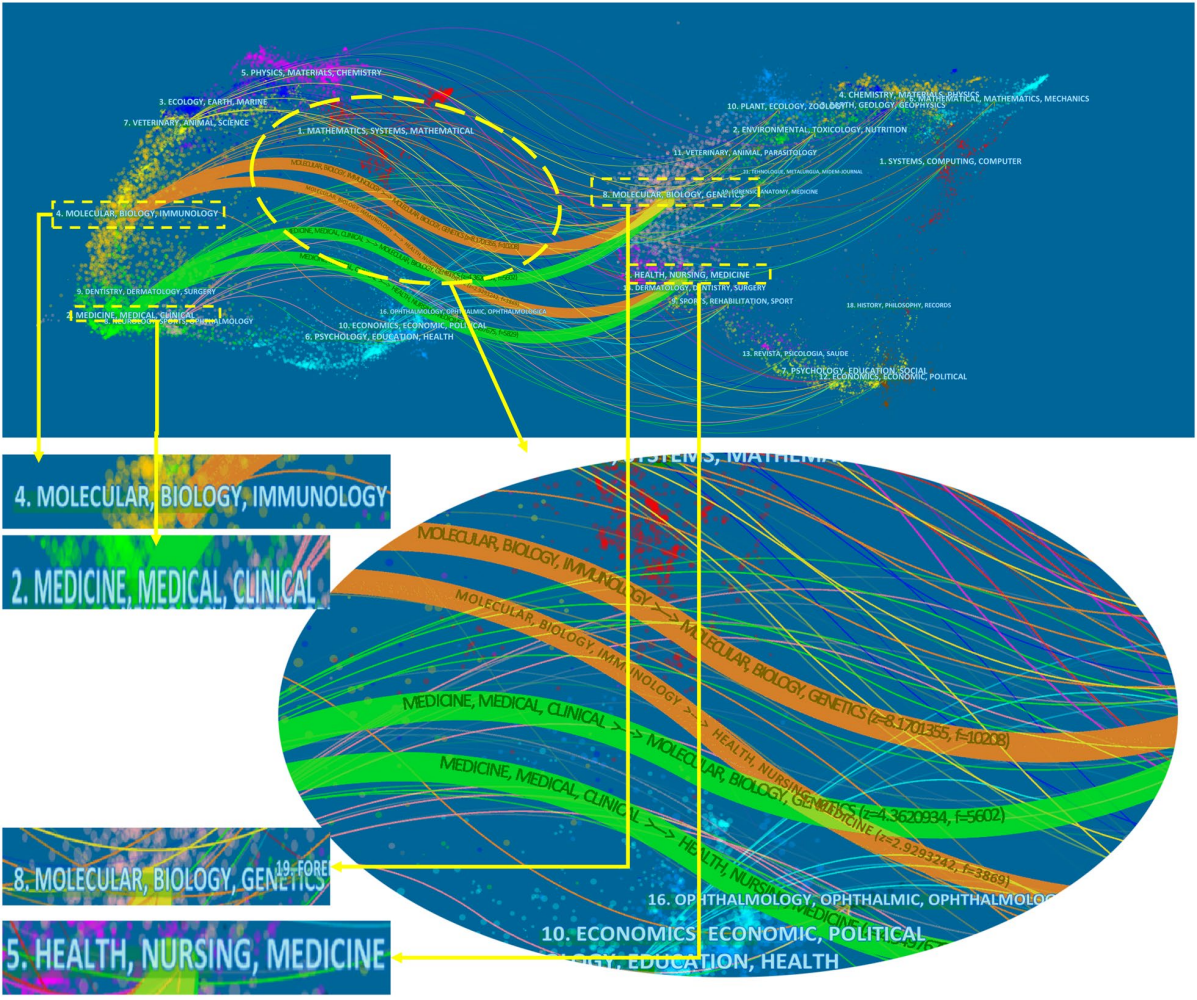
The dual-map overlay shows the distribution of publications related to circadian rhythm research in CVD. This graphical representation consists of major clusters, which are denoted by terms extracted from the titles of journals in the corresponding clusters. The journal in which a source article is published is called a “citing journal”. The journal in which a reference is published is called a “cited journal”. Notably, each spline curve depicted in the overlay originates from a citing journal located on the left-hand side of the base map and terminates at a cited journal situated on the right-hand side of the base map

856 journals published the articles on circadian rhythm in CVD. The top 10 journals with the highest TLCS are shown in Table 4. About 14.41% (*n* = 303) of the articles were published in these journals. The Chronobiology International (TLCS = 467) was the most prolific journal, followed by the Circulation (TLCS = 327) and the Circulation Research (TLCS = 226). Four main citation pathways were depicted in the dual-map overlay, the right for cited journals and the left for citing journals (the journal in which a source article is published is called a “citing journal”. A reference cited by a source article is called a “cited article”) (Fig. 5). The dual-map provides an understanding of the past development direction of the field of circadian rhythm in CVD [40]. The citing publications were mostly published in journals in the field of molecular biology, immunology, medicine, and medical clinical, whereas most of the cited publications were published in journals in the field of molecular biology, genetics, health nursing, and medicine.

**Table 4:** The top 10 core journals related to circadian rhythm in CVD

Rank	Source	Publications (*n*)	TLCS	TGCS	Average citation	2021 IF
1	Chronobiology International	135	467	3371	24.97	3.749
2	Circulation	19	327	2031	106.89	39.922
3	Circulation Research	12	226	1547	128.92	23.218
4	Hypertension	29	164	2615	90.17	9.897
5	American Journal of Physiology-heart and Circulatory Physiology	27	138	1197	44.33	5.125
6	Proceedings of The National Academy of Sciences of The United States of America	11	108	1173	106.64	12.779
7	Nature	2	98	1580	790.00	69.504
8	Sleep Medicine Reviews	15	97	1366	91.07	11.401
9	Hypertension Research	32	93	739	23.09	5.525
10	Journal of Hypertension	21	87	702	33.43	4.776

### Analysis of keywords

**Fig. 6:**
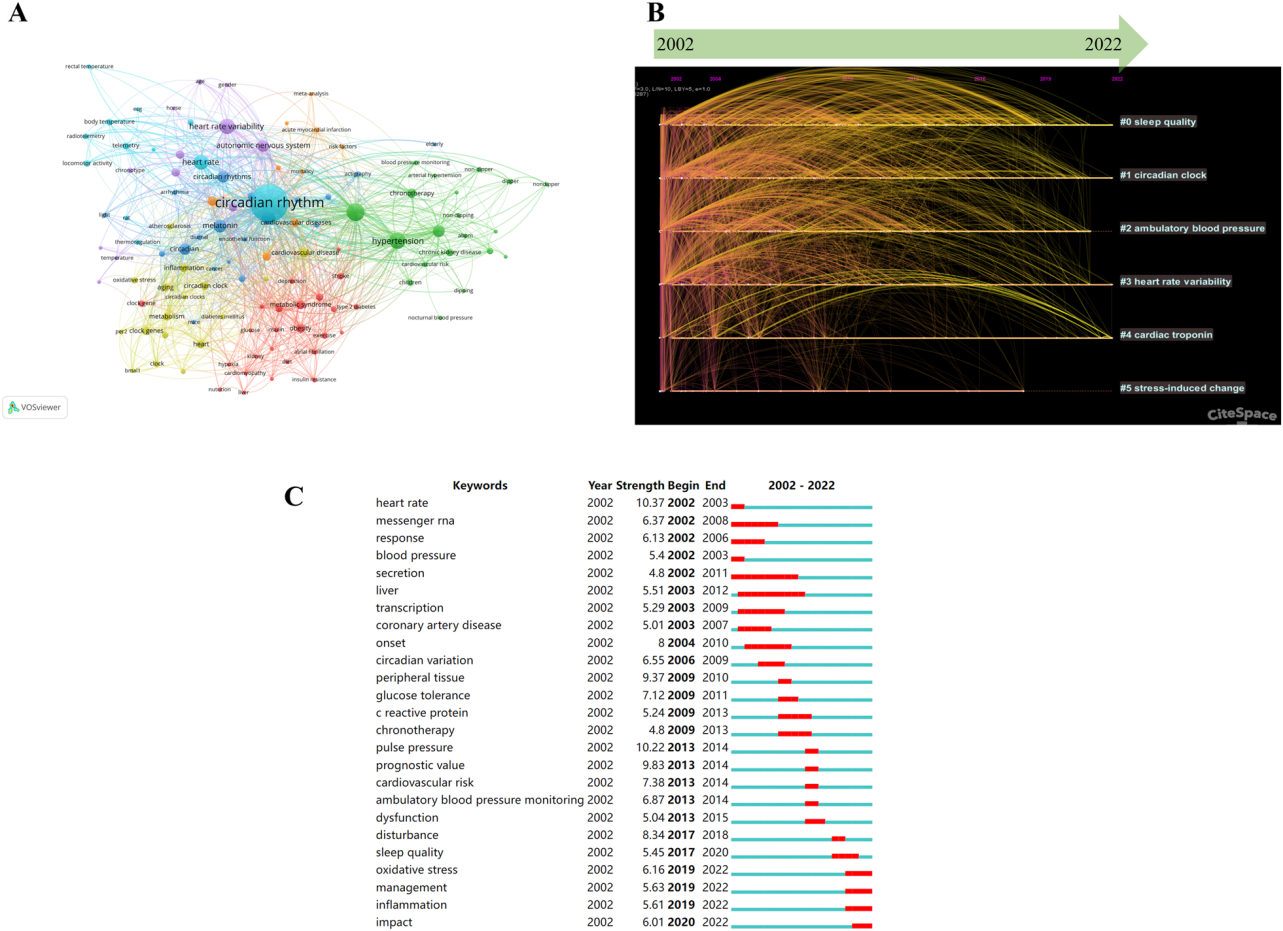
Visualization of keyword analysis. **A** The word cloud. **B** Timeline distribution of cluster analysis of keywords. **C** The top 25 keywords with the strongest bursts

The article’s basic content comprised keywords. Keyword analysis can be used to investigate research hotspots and frontiers in a field. During data collection, we collected 6548 keywords, and a cluster analysis produced 6 clustering outcomes (Table 5). The co-occurrence analysis of keywords revealed the primary topic in the field of circadian rhythm in CVD by presenting the keywords with a high frequency. The “circadian rhythm” was the most frequent keyword and held the central position in the network map (Fig. 6A). Figure 6B displays a screenshot of the timeline view, showcasing clusters arranged horizontally along timelines. The label for each cluster is positioned at the end of its corresponding timeline. The “cardiac troponin” (mean year = 2013) and the “stress-induced change” was the earliest (mean year = 2006) are the latest and earliest subjects, respectively. Moreover, burst keywords have been detected, which refers to keywords with a strong increase in frequency. The “strength” represents the burst extent. The “Begin” and “End” described the duration of the surge. A total of 82 keywords were extracted by keyword burst analysis, the top 25 of which are shown in Fig. 6C. The keyword “oxidative stress” had the highest burst strength in recent years.

**Table 5:** Keyword cluster analysis of circadian rhythm in CVD

Cluster ID	Size	Silhouette	Mean year	Top terms	Log (likelihood ratio)
#0	153	0.59	2012	Sleep quality	2374.76
#1	136	0.70	2009	Circadian clock	2687.78
#2	124	0.69	2009	Ambulatory blood pressure	4084.14
#3	120	0.59	2009	Heart rate variability	1731.10
#4	51	0.82	2013	Cardiac troponin	1599.12
#5	44	0.74	2006	Stress-induced change	439.02

### Co-cited references

**Fig. 7:**
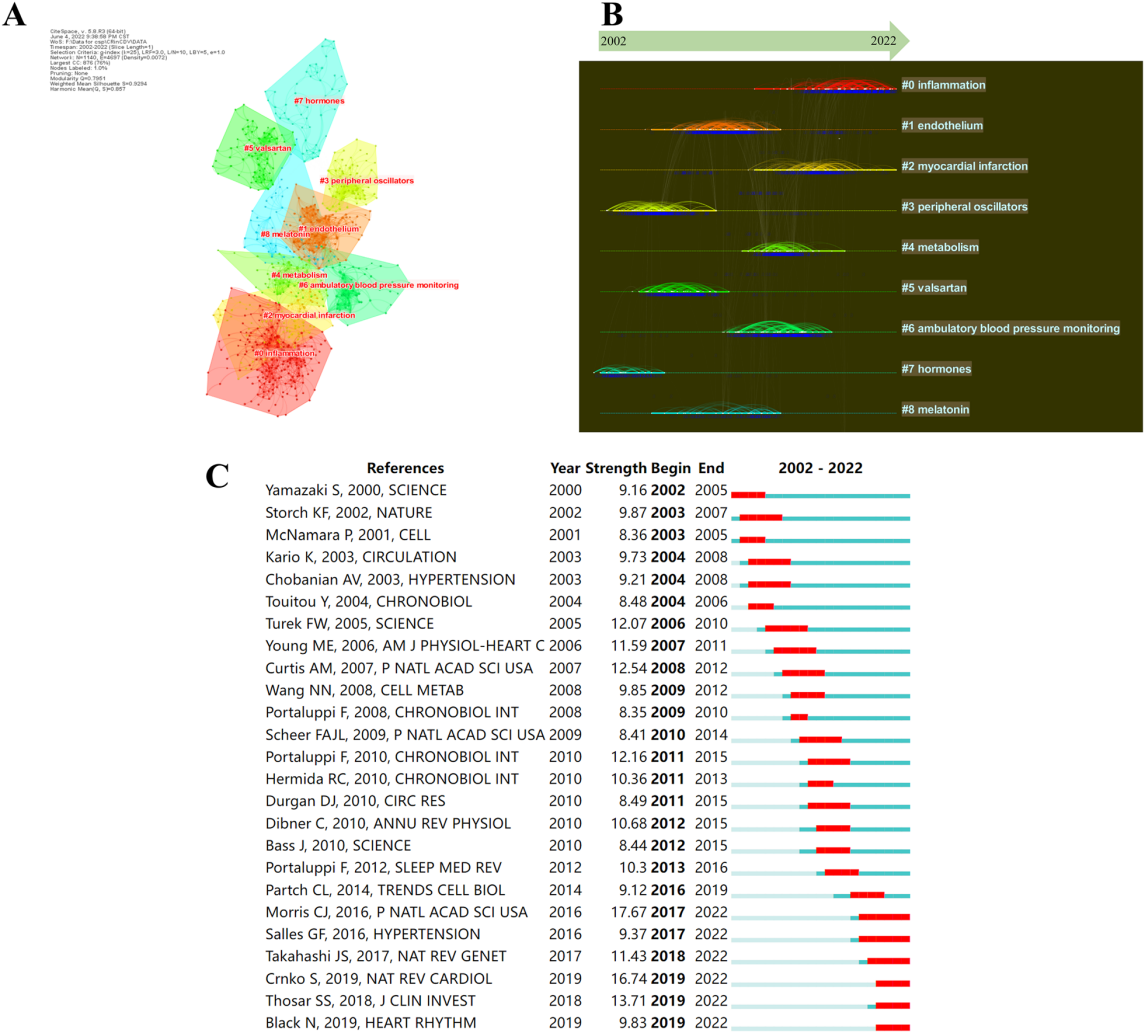
Visualization of co-cited reference analysis. **A** Cluster analysis of co-cited references. **B** Timeline distribution of the top nine clusters. **C** The top 25 references with the strongest citation bursts

When two publications were cited by a third document at the same time, the two documents are said to have a co-citation relationship; the higher the co-citation frequency, the closer their academic link and “distance”. We classify the publications in the discipline field based on this "distance" using the statistical approach of cluster analysis and visually identify and assess the segmentation in the discipline field using a graphical representation. The top 10 highest TLCS references included 2 reviews and 8 research articles (Table 6). Storch et al., authors of the article with the highest TLCS, found through a comparative analysis of mouse liver and heart in vivo that peripheral circadian gene regulation is extensive. They discovered marked differences in the distribution of circadian phases between the two tissues and only a few genes with circadian regulation in both [41]. Citespace was utilized to construct a visual network of co-cited references and a cluster analysis of documents was conducted. A total of 15 clusters were found, the modularity *Q* was 0.7951, and the mean silhouette value was 0.9294. There were nine clusters with the highest size values found (Table 7; Fig. 7A), which include “inflammation”, “endothelium”, and “myocardial infarction”, among others. We also designed a timeline view for clusters (Fig. 7B). We found that “hormones” is an early field of circadian rhythm in CVD (mean year = 1998). However, “inflammation” and “myocardial infarction” are the current hot topics (mean year = 2016/2014). Finally, a reference burst was performed from the co-citation investigation by Citespace. Figure 7C displays the top 25 references with the strongest citation bursts, which shows the most representative references in terms of burst strength, burst duration, and burst time. The team of Morris (2016) exhibited the highest burst strength. In their article, they demonstrated that circadian misalignment alone increases blood pressure and inflammatory markers [42].

**Table 6:** The top 10 papers with the highest TLCS

Rank	First author	Journal	Year	2021 IF	Cluster	TGCS	TLCS
1	Storch KF	Nature	2002	69.504	#3	1122	91
2	Curtis AM	Proceedings of The National Academy of Sciences of The United States of America	2007	12.779	#3	245	80
3	Viswambharan H	Circulation	2007	39.922	#1	145	53
4	Durgan DJ	American Journal of Physiology-heart and Circulatory Physiology	2005	5.125	#3	115	51
5	Durgan DJ	Circulation Research	2010	23.218	#1	178	48
6	Guo YF	American Heart Journal	2003	5.099	#3	111	46
7	Anea CB	Circulation	2009	39.922	#1	178	45
8	Portaluppi F	Sleep Medicine Reviews	2012	11.401	#1	169	42
9	Otto ME	Circulation	2004	39.922	#5	186	40
10	Westgate EJ	Circulation	2008	39.922	#1	95	39

**Table 7:** The top nine clusters of co-cited references with the highest size

Cluster ID	Size	Silhouette	Mean year	Top term	Log (likelihood ratio)
#0	205	0.879	2016	Inflammation	19.75
#1	118	0.898	2006	Endothelium	16.75
#2	87	0.928	2014	Myocardial infarction	10.89
#3	83	0.961	2001	Peripheral oscillators	17.92
#4	73	0.924	2011	Metabolism	8.01
#5	72	0.971	2003	Valsartan	11.99
#6	69	0.977	2010	Ambulatory blood pressure monitoring	43.83
#7	53	0.970	1998	Hormones	7.75
#8	49	0.924	2007	Melatonin	20.66

## Discussion

This study used multiple softwares to perform a bibliometric analysis of the global scientific outputs published related to the circadian rhythm in CVD from 2002 to 2022. The records from the WoSCC were examined from a variety of perspectives, with the results provided in tables and knowledge network maps. The results revealed that annual publications on circadian rhythm in CVD are on the rise. These studies prompt that circadian rhythm has taken an important role in the research of CVD.

The United States is at the forefront of promoting research on circadian rhythms in CVD, with six of the top 10 most productive institutions based there. Harvard University, which published 39 articles, cited 4244 times, was the main representative, being the most cited institution [41]. Hermida et al. from Universidade de Vigo contributed with most of the publications (published 34 articles, cited 1797 times), and with a long-term research focused on chronotherapy and risk of cardiac and cardiovascular risk [43, 44]. Simultaneously, a clinical trial from this group showed a provocative result, the authors reported that giving anti-hypertensives in the evening rather than the morning reduced the incidence of major cardiovascular events by 45% [45]. But as we found in the visual analysis, the existing data are not enough to support the large-scale promotion of their projects in clinical practice [46]. Concurrently, this seemingly ideal finding has garnered significant attention and, thus, has been subject to scrutiny [47].

Chronobiology International, in particular, had by far the highest number of articles published and citations among the top 10 core journals, indicating that this journal was the most popular journal for scholars who studied the field of circadian rhythm in CVD. In recent years, Chronobiology International has mainly focused on research exploring the relationship between circadian rhythm and diseases. For example, a recent article published in Chronobiology International found that in adults, a blunted rest-activity circadian rhythm is linked to higher white blood cell-based inflammatory indices, implying that lifestyle interventions aimed at restoring circadian rhythm could be a unique way to promote overall health [48]. In terms of several publications, Hypertension Research ranked second among the top 10 journals. This demonstrated that it is also appealing to researchers in this field. Hypertension Research has mainly focused on research discussing the correlation between hypertension and circadian rhythms. A recent study published in Hypertension Research discovered that increased overnight systolic blood pressure adds to the influence of high NT-proBNP levels on the risk of CVD [49]. Molecular biology and medicine are two interrelated fields that share an interest in investigating the fundamental biological mechanisms of life. Molecular biology explores the underlying molecular processes that drive biological activity, while medicine applies scientific knowledge to the prevention, diagnosis, and treatment of disease. According to the result of the dual-map overlay, the significant interest in investigating the link between circadian rhythms and cardiovascular health among researchers in the fields of molecular biology and medicine is understandable. It highlights the fact that researchers are actively exploring the molecular mechanisms that underlie this connection and are striving to develop new interventions and treatments for CVD based on circadian biology [50].

Following the cluster analysis of co-cited references, “hormones” was identified as an early hotspot in the research of circadian rhythms in CVD. After retrieving the publications in this cluster (#7), a study with the highest TGCS on the relationship between leptin and sleep was located, which demonstrated that sleep modulation is a major element of the neuroendocrine control of appetite [51]. Subsequently, Scheer et al. discovered that taking melatonin regularly lowered systolic and diastolic blood pressure during sleep [52]. Similarly, Tutuncu et al. discovered a negative correlation between nocturnal melatonin levels and the degree of nocturnal systolic blood pressure drop [53]. In addition, “secretion” had the strongest burst strength and longest bursts times in the early years, so we retrieved it from the database and found that almost all of the highest TGCS research had a focus on the relationship between the circadian rhythms and hormones—such as leptin [54], melatonin [55], cortisol [56], insulin [57] and oxytocin [58]. Therefore, it is revealed that the studies frequently focused on the influence of circadian rhythm on hormone levels in the early stage of this field.

With the advancement of circadian rhythms in CVD, certain developing study domains are progressively gaining attention from researchers. It is well known that atherosclerosis involves an ongoing inflammatory response [59], and coronary atherosclerosis is the main cause of myocardial infarction [60]. This also explains why the timeline view of references showed that “myocardial infarction” and “inflammation” have attracted the attention of scholars in recent years. According to representative burst references, a clinical study demonstrated that circadian misalignment per se increases blood pressure and inflammatory markers, which will increase hypertension, inflammation, and the risk of CVD [42]. Therefore, circadian rhythmicity appears to have an important role in atherosclerosis by affecting inflammatory processes underlying atherosclerosis, according to growing research [61–65]. All together, these findings suggest that circadian rhythmicity is becoming increasingly important in CVD. Based on these recent discoveries, it is evident that circadian rhythmicity is already a viable target for therapeutic strategies in CVD [66–68]. There have been inconsistencies in clinical evidence of circadian-based therapies for CVD, however, the recent TIME trial suggested that chronotherapy has no negative outcomes [69].

In conclusion, this bibliometric analysis has provided an overview of the research landscape related to circadian rhythm in CVD from 2002 to 2022. The findings reveal the importance of circadian rhythmicity in CVD research and highlight the potential for incorporating it into therapeutic strategies. Furthermore, researchers should focus on investigating how to effectively integrate circadian rhythm as a therapeutic treatment in clinical applications, making it more affordable and accessible for patients. By addressing these limitations and focusing on the potential benefits of circadian rhythmicity in CVD, our study contributes to the ongoing efforts to understand and develop novel therapeutic strategies in the field of CVD.

## Limitation

Although bibliometric analysis provides greater insight into research topics and trends than traditional assessments, it has several limitations. To begin with, this article excludes non-English literature, which might introduce bias. Additionally, the data used in this study were obtained solely from the WoSCC database due to the reliability of the publications and citations. We cannot perform relevant analyses, such as co-citation analysis, on PubMed or other databases due to software limitations (lack of information on the references), which further contributes to the study bias. Consequently, the local dataset we downloaded from the WoSCC may have fewer articles and journals compared to other databases like PubMed, resulting in less comprehensive study findings.

Moreover, we examined the characteristics of the data we collected to highlight the most important aspects. As a result, some information may be overlooked. Lastly, most of the results in this study are based on a machine algorithm, which significantly lacks human selection. (For example, the selection of terms in cluster analysis will cause readers a certain degree of confusion.) Due to the sensitivity of machine algorithms, several emerging research areas related to circadian rhythmicity in CVD may not have been included.

## Data Availability

Not applicable.
